# CTCF delimits a DNA methylation transition at the intergenic region between the embryonic and adult α-globin genes

**DOI:** 10.1080/15592294.2026.2711190

**Published:** 2026-08-03

**Authors:** Gustavo Tapia-Urzúa, Hober Nelson Núñez-Martínez, Sylvia Garza-Manero, Martin Franke, Carlos Alberto Peralta-Alvarez, Ángel Josué Cerecedo-Castillo, Georgina Guerrero, José Luis Gómez-Skarmeta, Félix Recillas-Targa

**Affiliations:** a Instituto de Fisiología Celular, Departamento de Genética Molecular, Universidad Nacional Autónoma de México, Ciudad de México, México; b Tecnologico de Monterrey, Escuela de Medicina y Ciencias de la Salud, Monterrey, México; c Centro Andaluz de Biología del Desarrollo (CABD), Consejo Superior de Investigaciones Científicas/Universidad Pablo de Olavide, Sevilla, Spain; d Unidad de Bioinformática y Manejo de la Información, Instituto de Fisiología Celular, Universidad Nacional Autónoma de México, Ciudad de México, México

**Keywords:** CTCF, chromatin accessibility, DNA methylation, gene silencing, erythropoiesis

## Abstract

Transcriptional activation of cell-type-specific genes is achieved by multiple mechanisms that guarantee chromatin accessibility at *cis*-regulatory elements and the long-range interactions between them. Generally, DNA methylation counteracts these processes and promotes gene silencing in vertebrates. CTCF is a conserved and essential multifunctional transcription factor relevant for establishing and maintaining chromatin architecture and accessibility, while its precise role in regulating DNA methylation remains to be determined. We focused on the highly abundant and previously studied erythroid-specific α^D^ gene (*HBAD*) to systematically investigate the role of CTCF binding on DNA methylation, chromatin accessibility, and gene expression using a chicken erythroid cell differentiation system. The perturbation of CTCF binding at the intergenic region between the embryonic π and the adult α^D^ gene resulted in DNA methylation propagation towards the α^D^ gene, which was accompanied by decreased chromatin accessibility, GATA-1 binding, and gene expression. Notably, chromatin conformation analyses revealed that CTCF binding enables α^D^ transcriptional activation independently of its architectural function. We observed a similar role of CTCF on DNA methylation and gene expression in the human orthologous gene *HBA2*. Our findings support a conserved role of CTCF in preventing DNA methylation spreading and gene silencing of cell-type-specific genes along differentiation.

## Introduction

The transcriptional regulation of cell-type-specific genes is governed by molecular mechanisms that control chromatin accessibility of *cis*-regulatory elements for transcription factor binding (CREs) [1, 2]. Among these, DNA methylation is a highly conserved mechanism [3, 4], where the covalent addition of a methyl group (–CH_3_) to the 5′ carbon of cytosine (5mC) within a CpG dinucleotide context is associated with transcriptional repression when occurring in high frequency at promoters and intergenic regions [5–8]. This silencing effect is mediated by two proposed mechanisms: 1) recruitment of methyl-binding proteins, including co-repressors and chromatin remodeling enzymes, that induce chromatin compaction, and 2) steric hindrance of transcription factors binding to their cognate motifs [3].

DNA methylation of promoters and other regulatory elements occurs dynamically during cell differentiation, which coincides with the binding of cell-type-specific transcription factors (TFs) and CTCF. It has been shown that CTCF, in particular, is required for establishing cell-type specific DNA methylation patterns at enhancers and for ensuring proper expression of target genes [7, 9–11]. Additional evidence supports the role of CTCF in inhibiting local DNA methylation at promoter regions and in facilitating gene transcription in cancer [12].

CTCF is a highly conserved 11-zinc-finger nuclear protein originally identified as an insulator-binding factor [13]. Insulators are DNA-protein complexes characterized by two main properties: 1) enhancer-blocking activity preventing aberrant promoter–enhancer interactions, and 2) barrier activity, which halts the spread of heterochromatin features [14–17]. More recently, the role of CTCF as a primary organizer of chromatin architecture has been extensively documented [18–21]. Both the insulator and architectural functions of CTCF have been proven essential for shaping chromatin structure and gene expression in several biological processes [22–26].

Erythropoiesis has emerged as a powerful model for studying *in vivo* and *in vitro* the mechanisms governing gene regulation during cell differentiation in metazoans, including DNA methylation, DNA-binding factors, and chromatin remodeling [27–29]. In this context, CTCF is required for cell proliferation and differentiation during erythropoiesis, from hematopoietic stem and progenitor cells (HSPC) to erythroblasts [30–34]. However, the impact of CTCF on chromatin accessibility, DNA methylation, and gene expression in later stages of erythropoiesis has been less explored, due to erythroid enucleation in mammals [35]. In this sense, avian erythrocyte maturation serves as a model for studying chromatin remodeling and gene regulation within an erythroid context where the nuclei are retained [36, 37].

In this study, we profiled genome-wide CTCF occupancy in chicken erythroblasts induced to terminal differentiation *in vitro* to identify dynamic binding sites associated with transcriptional activation. We integrated chromatin accessibility and transcriptome analysis to identify a subset of intergenic and promoter-proximal regions targeted by CTCF, which coincide with upregulated genes during erythroid maturation. Using the previously studied chicken alpha globin α^D^ gene (*HBAD*) as a model, we demonstrate that CTCF binding in the intergenic region between the embryonic π and adult α^D^ genes is essential to prevent the spread of DNA methylation towards the α^D^ gene in differentiating cells. This binding also promotes local chromatin accessibility and enables transcriptional activation. Notably, this CTCF blocking effect occurs independently of its architectural function. We further explored this mechanism on the human orthologous *HBA2* gene, revealing a conserved barrier function of CTCF in human cells. Our study supports a role of CTCF in preventing DNA methylation to ensure proper gene activation during erythroid maturation.

## Materials and methods

### Cell culture

HD3 cells were grown as previously described [38]. To induce erythroid differentiation, HD3 cells were treated with H-7 dihydrochloride (20 μM; Sigma) in culture medium containing 8% FBS (MultiCell), 2% chicken serum (Gibco), 1% penicillin/streptomycin (Gibco), and 10 mM HEPES, pH 8.0, in a 1% CO_2_ atmosphere at 42°C for 24, 48, and 72 h.

### Chromatin immunoprecipitation followed by qPCR (ChIP-qPCR)

Chromatin immunoprecipitation (ChIP) for CTCF, VEZF1, GATA-1, and YY1 was performed as previously described with some modifications [39]. Briefly, 2 × 10^7^ HD3 cells/mL and 2 × 10^6^ K562 cells/mL were cross‑linked in 1X PBS (phosphate-buffered saline) with 1% formaldehyde for 10 min, quenched with 0.125 M glycine for 5 min, and washed twice with 1X PBS. Cells were resuspended in lysis buffer (10 mM Tris-HCl pH 7.5, 10 mM NaCl, 0.3% NP-40, and protease inhibitors [PIK]) for 30 min at 4°C. The nuclear pellet was lysed in nuclear buffer (50 mM Tris-HCl pH 7.5, 10 mM EDTA, 1% SDS, and PIK). Chromatin was sonicated (Bioruptor; 30 cycles of 9.9 sec on and 9.9 sec off at 35% amplitude, 300 sec total). For each immunoprecipitation, 50 μg chromatin was diluted 1:5 in dilution buffer (1% Triton X-100, 2 mM EDTA, 20 mM Tris-HCl, 150 mM NaCl, and PIK), precleared with protein G/A beads (50 μL for 2 h), then incubated overnight at 4°C with the corresponding antibodies as follows: 2 μg IgG (Millipore 12–370), 4 μg GATA-1 (Santa Cruz sc-13053), 5 μg YY1 (Diagenode C15410345), 5 μg VEZF1 (Abcam ab68278), 5 μg CTCF (Millipore 07–729), and 10 μL CTCF [39]. Next, 30 μL of protein G/A beads were added and incubated for 2 h at 4°C. Beads were washed four times with buffer I (0.1% SDS, 1% Triton X-100, 2 mM EDTA, 20 mM Tris-HCl, 150 mM NaCl, and PIK) and once with buffer II (0.1% SDS, 1% Triton X-100, 2 mM EDTA, 20 mM Tris-HCl, 500 mM NaCl, and PIK). Beads were eluted in 300 μL of elution buffer (1% SDS and 100 mM NaHCO_3_). Decross‑linking was performed as follows: 37°C for 1 h with RNase A (Ambion) and 65°C for 4 h with proteinase (NEB). DNA was extracted with phenol:chloroform:isoamyl alcohol (Invitrogen). The aqueous phase was retrieved and the DNA was precipitated by adding 1 M ammonium acetate, glycogen (Roche), ethanol, and incubated for 2 h at −70°C. The DNA pellet was washed with 70% ethanol and resuspended in 30 μL nuclease-free water. For ChIP-qPCR, 1 μL of retrieved DNA and input was used for real-time PCR (qPCR) using iTaq Universal SYBR Green Supermix (Bio-Rad). Oligonucleotides used for ChIP-qPCR are listed in Supplementary Table S1.

### Chromatin immunoprecipitation followed by sequencing (ChIP-seq)

ChIP-seq for CTCF was performed by ChIPmentation as previously described [40, 41]. Briefly, 2 × 10^7^ HD3 cells/mL were cross-linked in 1% formaldehyde for 10 min in 1X PBS. The cross-linking reaction was quenched with 0.125 M glycine for 5 min. The cells were washed twice with 1X PBS, resuspended in cell lysis buffer (10 mM Tris-HCl pH 7.5, 10 mM NaCl, and 0.3% NP-40, and PIK), and incubated for 30 min at 4°C. The nuclear pellet was resuspended in 333 µl of nuclear lysis buffer (50 mM Tris-HCl pH 7.5, 10 mM EDTA, 1% SDS, and Complete protease inhibitors cocktail [Roche]), and incubated for 5 min on ice. The nuclear lysate was diluted with 667 µl of ChIP dilution buffer (16.7 mM Tris-HCl pH 7.5, 1.2 mM EDTA, 167 mM NaCl, 0.01% SDS, and 1.1% Triton-X100). Then, chromatin was sonicated in a Covaris M220 bioruptor (duty cycle 10%, PIP 75 W, 100 cycles/burst, 10 min) and centrifuged for 5 min at 4°C. The supernatant was used for ChIP. For each immunoprecipitation, 250 µl of chromatin were used and incubated with 10 µl of anti-CTCF and incubated by rotation overnight at 4°C. The next day, 20 µl of protein G Dynabeads (Invitrogen) were washed twice with ChIP dilution buffer and resuspended in 50 µl of ChIP dilution buffer. Immunoprecipitated chromatin was incubated with washed beads for 1 h by rotation at 4°C and washed twice with wash buffer 1 (20 mM Tris-HCl pH 7.5, 2 mM EDTA, 150 mM NaCl, 1% SDS, and 1% Triton-X100), wash buffer 2 (20 mM Tris-HCl pH 7.5, 2 mM EDTA, 500 mM NaCl, 0.1% SDS, and 1% Triton-X100), and 10 mM Tris-HCl pH 8.0, using a cold magnet (Invitrogen). Then, beads were resuspended in 25 µl of TAGmentation reaction mix (10 mM Tris-HCl pH 8.0, 5 mM MgCl_2_, and 10% w/v dimethylformamide) with 1 µl of Tn5 enzyme (Diagenode) and incubated for 1 min at 37°C. The TAGmentation reaction was put in the cold magnet, and the supernatant was discarded. Beads were washed twice with wash buffer 1 and TE buffer (10 mM Tris and 1 mM EDTA) and eluted twice for 15 min in 100 µl of elution buffer (50 mM NaHCO_3_ pH 8.8 and 1% SDS). The eluted chromatin was decross‑linked by adding 10 µl of 4 M NaCl and 1 µl of 10 mg/ml Proteinase K and incubating at 65°C for 6 h. DNA was purified using the MinElute PCR Purification Kit (Qiagen) and eluted in 20 µl of TE buffer. Library preparation was performed as previously described for ATAC-seq [40, 41]. Libraries were pooled and sequenced on a HiSeq 2500 as paired-end (20 × 10^6^ reads per sample) 150 bp reads.

### ATAC-seq

ATAC-seq assays were performed as previously described with minor modifications [40, 42]. Briefly, 5 × 10^5^ HD3 cells were lysed in 50 µl of lysis buffer (10 mM Tris-HCl pH 7.4, 10 mM NaCl, 3 mM MgCl_2_, 0.1% NP-40, and 1X Complete protease inhibitors cocktail) by pipetting up and down. The whole-cell lysate was incubated on ice for 10 min and centrifuged for 10 min at 500 g at 4°C. Supernatant was removed and the nuclear extract was resuspended in 50 µl of the transposition reaction containing 2.5 µl of Tn5 enzyme and TAGmentation Buffer (10 mM Tris-HCl pH 8.0, 5 mM MgCl_2_, and 10% w/v dimethylformamide), and incubated for 30 min at 37°C. Immediately, DNA was purified using the MinElute PCR Purification Kit and eluted in 20 µl of TE buffer (10 mM Tris and 1 mM EDTA). Libraries were generated by PCR amplification using the KAPA HiFi HotStart PCR Kit (Roche), pooled and sequenced in a HiSeq 2500 as paired-end (30 × 10^6^ reads per sample) 150 bp reads.

### ChIP-seq and ATAC-seq data analysis

Raw reads were trimmed for adapters and low-quality reads using TrimGalore. Reads were aligned to the chicken genome assembly galGal6 (GRCg6a) using Bowtie 2 with default parameters [43]. BAM files were filtered as follows: properly paired reads were included; reads marked as secondary alignments, PCR duplicates, and low-quality mapped reads were removed using NGSUtils [44]. Peak calling for ATAC-seq data was performed without building the shifting model, and a peak detection based on FDR < 0.05 was performed using MACS2 [45]. For ChIP-seq, peak calling was performed by building the shifting model, setting the lower mfold bound to 5, the upper mfold bound to 50, the band width to compute fragment size to 300, and a peak detection based on *p*-value < 0.01. Peaks overlapping in each condition were selected for raw counting using Intervene [46]. Aligned raw counts over peaks for each replicate and condition were generated using featureCounts [47]. Differentially CTCF bound regions between differentiated and undifferentiated HD3 cells were identified as follows: FDR < 0.05 and log2FC ≥ 1 using edgeR and R [48]. Differentially accessible regions from ATAC-seq data were identified by using DiffBind (FDR < 0.05) [49]. Normalized ChIP-seq and ATAC-seq signals were generated by merging BAM files for each replicate using NGSUtils and deepTools2 [50]. Heatmaps and profile plots were generated using deepTools2. Peak annotation was conducted by ChIPSeeker [51]. Motif analysis was performed using HOMER [52]. Gene ontology was conducted by Metascape [53].

### Public data analysis

Raw reads from WGBS in K562 cells were retrieved from GSE50610 [54]. The reads were aligned to the human genome assembly hg19 (GRCh37) and used to generate a signal track using Bismark [55]. ChIP-seq and ATAC-seq data processing was performed as mentioned previously. Hi-C matrices were generated by converting validPairs files using HiC-Pro and visualized through Juicebox [56, 57]. Genomic data visualization was performed using IGV [58].

### UMI-4C

The circular chromosome conformation capture with the unique molecular identifier (UMI-4C) protocol was performed as previously described with minor modifications [59]. Two replicates per condition were performed. Briefly, 2 × 10^7^ cells were cross-linked with 1 mL of 1X PBS containing 1% paraformaldehyde for 10 min at RT by rotation. The cross‑linking was quenched with 0.125 M glycine for 5 min on ice. The cell pellet was washed twice with 1X PBS at 4°C. The nuclear pellet was obtained by adding 1 mL of cell lysis buffer (50 mM Tris pH 7.5, 150 mM NaCl, 5 mM EDTA, 0.5% NP-40, 1.15% Triton X-100, and 1X complete protease inhibitors) and incubating on ice for at least 10 min. Then, the cells were centrifuged for 5 min at 750 g at 4°C and washed with 1X PBS. The nuclear pellet was resuspended in 100 µL 0.5% SDS and incubated for 10 min at 62°C without shaking. To quench the remaining SDS, 292 µL water and 50 µL 10% Triton X-100 were added to each sample and incubated for 15 min at 37°C. The chromatin was digested with 400 units of DpnII (NEB, R0543) in 50 µL of 10X restriction buffer at 37°C for 4 h with 86 g shaking. Next, 400 units of DpnII were added in 450 µL of 1X RE buffer at 37°C overnight. DpnII was inactivated at 65°C for 20 min. Biotin fill-in and proximity ligation were performed with Klenow (NEB, M0210L) and T4 DNA ligase (NEB, M0202L), respectively. The nuclear pellet was centrifuged for 10 min at 600 g at 4°C. Then, 800 µL of the supernatant was removed, and 230 µL of Proteinase K buffer, 20 µL of Proteinase K (10 mg/mL), and 50 µL of 10% SDS were added and incubated for 30 min at 55°C. Then, 40 µL of 4 M NaCl was added, and incubated at 65°C overnight with 52 g shaking. The next day, 5 µL of RNase A (10 mg/mL) was added, followed by incubation for 30 min at 37°C at 52 g shaking. Then, 20 μL of Proteinase K (10 mg/mL) was added and incubated for 2 h at 55°C at 52 g shaking. DNA was purified with phenol:chloroform:isoamyl alcohol (Invitrogen). The aqueous phase was retrieved following DNA precipitation by adding 1 M ammonium acetate, glycogen (Roche), and cold ethanol. The DNA pellet was washed with 70% ethanol and resuspended in 100 µL of 10 mM Tris-HCl pH 7.5. Subsequently, 7 µg of purified DNA was sheared using a Covaris M220 bioruptor. The DNA was purified using AMPure XP beads (Agencourt, A63881). DNA was resuspended in 300 µL of water. Biotin-labeled DNA was bound to Dynabeads MyOne Streptavidin C1 beads using 5 µL of beads per 1 µg DNA by following the manufacturer’s instructions to perform the pull-down process. Retrieved beads were eluted in 50 µL water. Next, 500 ng of DNA was attached to the streptavidin beads following sequential incubation using end-repair mix (1× T4 Ligase Buffer, 0.5 mM dNTP mix, 0.12 U/µL T4 DNA Polymerase [NEB, M0203], and 0.05 U/µL Klenow [NEB, M0210]), A-tailing mix (1X NEB buffer 2, 0.5 mM dATP, and 0.25 U/µL Klenow, exo- [NEB, M0212]), and CIP (NEB, M0290). The samples were indexed by ligating TruSeq Illumina adapters by incubating DNA-bound beads in an adapter ligation mix (1× T4 Ligation Buffer, 5% PEG-4000, and 0.3 U/µL T4 DNA ligase [Thermo Fisher, EL0011]) at RT for 2 h. The DNA-bound beads were denatured to remove the non-ligated strand of the adaptor. After washing, the DNA-bound beads were resuspended in 20 µL of nuclease-free water. Then, 200 ng of DNA-bound beads were used for library preparation by nested PCR. Three PCRs (20 cycles) were performed and then pooled for AMPure bead purification for each sample for the first PCR. In addition, for the second PCR (10 cycles), 4 PCRs were performed for each sample and then pooled for size selection for DNA fragments between 200 and 700 bp using AMPure beads. Libraries were pooled and sequenced using DNBSEQ technology to produce 50 bp paired-end reads and approximately 1–4 million raw sequencing read pairs for the α^D^ viewpoint for each sample. For the UMI-4C data analysis, FASTQ files were processed using UMI4Cats [60]. For the visualization of the chromatin contact profiles, we used the default parameters. The oligonucleotides used for UMI-4C are listed in Supplementary Table S1.

### CRISPR-Cas9 genome editing

The single-guide RNAs (sgRNAs) used in this study were designed using the CRISPOR web tool (http://crispor.gi.ucsc.edu/) [61] with the galGal6 and hg19 genome annotations. Oligonucleotides for the guides were cloned into pSpCas9(BB)-2A-Puro (PX459) V2.0 (a gift from Feng Zhang, Addgene #62988), as previously described [62]. The integrity of the cloned sgRNA was evaluated by Sanger sequencing. Five hundred nanograms of plasmid were transfected into 5 × 10^5^ HD3 cells using Lipofectamine 2000 Reagent (Invitrogen) according to the manufacturer’s protocol. Next, 1 μg/mL of puromycin (Sigma) was added to the transfected cell culture after 24 h of plasmid transfection. After 7 days of puromycin selection, an aliquot of cells was used for DNA extraction by phenol–chloroform and PCR genotyping using specific primers spanning the desired deletion. 1 × 10^6^ of the pool mutant cells was used for serial dilutions in a 96-well plate containing media. Single clones were identified by microscopy after 2 weeks and expanded for subsequent genotyping by PCR. The deletions of the mutant cell clones were further characterized by cloning the PCR fragments into the pGEM-T Easy vector (Promega) and confirmed by Sanger sequencing. The oligonucleotides used for the CRISPR-Cas9 genome editing assay are listed in Supplementary Table S1.

### RNA isolation and RT-qPCR

Total RNA was isolated using TRIzol Reagent (Invitrogen) according to the manufacturer’s instructions with minor modifications. Cells were pelleted and resuspended in TRIzol Reagent and incubated for 10 min by rotation. Chloroform (Invitrogen) was added and mixed by rotation for 10 min. The aqueous phase was retrieved by centrifugation for 10 min at 4°C. The RNA pellet was obtained by adding 2-propanol (Invitrogen) and centrifuged for 10 min at 4°C. RNA was washed twice with 75% ethanol and resuspended in nuclease-free water. RNA was used for RT-qPCR to determine gene expression using the KAPA SYBR FAST One-Step Kit (Roche). *RPL27* was used as an endogenous control. RT-qPCR data were analyzed by the ΔΔCt method [63]. The oligonucleotides used for RT-qPCR are listed in Supplementary Table S1.

### RNA-seq data analysis

Public RNA-seq datasets used in this study were retrieved as follows: fibroblasts and erythroblasts (GSE206193); undifferentiated and differentiated HD3 (GSE76573). Raw reads were mapped to the chicken genome assembly galGal6 (GRCg6a) using STAR with default parameters [64]. Gene-level read counts were assessed using featureCounts with NCBI RefSeq annotation release 104 [47]. Differential gene expression analysis was performed using edgeR with a threshold of FDR < 0.05 and log2FC ≥0.5 [48]. Mapped reads of each replicate were merged and filtered as follows: properly paired reads were included; reads marked as secondary alignments, PCR duplicates, and low-quality mapped reads were removed using NGSUtils. Read-density files were generated from the filtered mapped reads using deepTools2 [50]. The volcano plot was generated using the ggplot2 and ggrepel packages in R.

### Sodium bisulfite DNA conversion and sequencing

Sodium bisulfite DNA conversion was performed as previously described with minor modifications [65]. Briefly, 3 μg of genomic DNA was incubated with 0.3 M NaOH at 37°C for 5 min, denatured at 95°C for 2 min, and chilled on ice. Sodium bisulfite pH 5.0 (Sigma) and hydroquinone (Sigma) were added at final concentrations of 2.6 M and 0.5 mM, respectively. The reactions were incubated at 50°C for 16 h in the dark. Non-reacting bisulfite was removed by column purification using the Wizard DNA Clean-Up System (Promega). Purified DNA was desulphonated with NaOH at a final concentration of 0.3 M and incubated at 37°C for 15 min followed by ethanol precipitation. DNA fragments of interest were PCR-amplified using the modified primers listed in Supplementary Table S1. A nested PCR was performed for the amplification of the α^D^ gene promoter DNA fragment. For chicken HD3 cells, three independent biological replicates were performed, yielding 8–10 sequenced clones per replicate. For human K562 cells, two independent biological replicates were performed. The number of sequenced clones per replicate was as follows: 10 and 14 clones for wild-type K562 (total *n* = 24), and 9 and 10 clones for the Δ11 mutant (total *n* = 19). PCR products were cloned into the pGEM-T Easy (Promega) and sequenced using T7 or SP6 sequence primers by Sanger sequencing.

### Knockdown assays

Short-hairpin RNAs (shRNAs) against CTCF and VEZF1 were designed using the online splashRNA (http://splashrna.mskcc.org/) [66]. Oligonucleotides corresponding to the shRNAs were aligned and cloned into the pTRIPZ vector (a gift from Ming Chen, Addgene plasmid #206981) [67]. Five hundred nanograms of plasmid were transfected into 5 × 10^5^ HD3 cells using Lipofectamine 2000 Reagent (Invitrogen) according to the manufacturer’s protocol. Cells containing the corresponding vector were selected with puromycin. To induce shRNA expression, cells were treated with 1 μg/mL of doxycycline for 72 h before the induction of differentiation and extraction of RNA. For CTCF knockdown in K562 cells, a control shRNA or an shRNA targeting CTCF cloned into the pTRIPZ vector (a gift from Danny Reinberg) was transfected, selected with puromycin, and induced with doxycycline as previously mentioned [68].

### Statistical analysis

Data represent the mean ± standard error (SEM) of at least two biological replicates. The significance (*p*-value) was determined by a two-tailed unpaired Student´s t-test using GraphPad Prism 9. *p*-values determined by Student´s t-test were not corrected for multiple comparisons. For box plots and profile plots, significance (*p*-value) was determined by a two-sided Mann-Whitney U test in R.

### Additional datasets used in this study

Public datasets were reanalyzed and used in this study as follows: RNA-seq for chicken fibroblasts and erythroblasts (GSE206193); undifferentiated and differentiated HD3 (GSE76573). ChIP-seq for CTCF (GSE51846) and RNAP II (GSE228694) in HD3. ATAC-seq for fibroblasts and erythroblasts (GSE206191). Hi-C for fibroblasts and erythroblasts (GSE206192). ChIP-seq, ATAC-seq, and RNA-seq in HSPC and erythroblasts (GSE131055). ChIP-seq, ATAC-seq, and RNA-seq in HUDEP-2 (GSE201822). RNA-seq for K562 (HRA009396 and GSE217759).

## Results

### CTCF is required for α^D^ gene activation

CTCF binding has been documented to function as a direct modulator of local DNA methylation in a cell-type-specific manner. This occurs primarily at distal regulatory regions (more than 1 kb from the TSS), where CTCF is required to maintain low DNA methylation levels, suggesting a protective role against methylation [7]. Additionally, it has been suggested that certain CTCF sites function as boundaries to prevent DNA methylation spreading [69]. However, this role remains controversial due to differences in the methods and models used to measure methylation dynamics [12,70].

**Figure 1.**
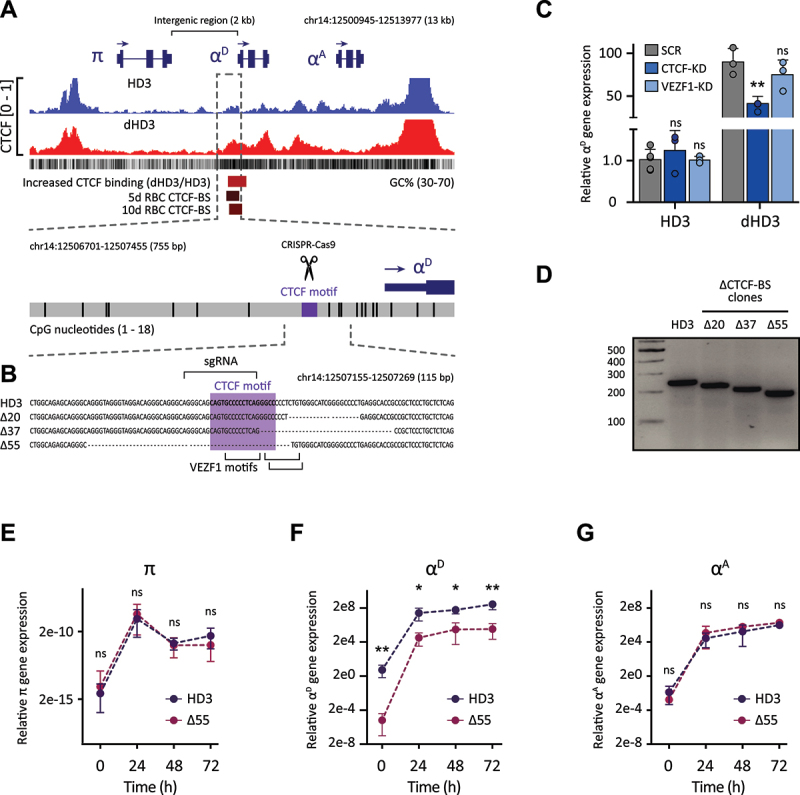
The CTCF-BS is critical for the transcriptional activation of α^D^. (A) Genomic snapshot showing the CTCF signal tracks for HD3 and dHD3 at the chicken α-globin locus. The inset boxes show CTCF peaks for dHD3, 5-day red blood cells (5d RBCs), and 10-day red blood cells (10d RBCs). A zoomed-in view of the intergenic region between the π- and α^D^-globin genes shows the position of the CTCF (purple box) motif deleted by CRISPR-Cas9. Vertical black lines represent the positions of CpG nucleotides along the intergenic region analyzed. (B) Multiple alignments showing the sequences of the ΔCTCF-BS clones (Δ20, Δ37, and Δ55) compared to HD3 cells. Sequences were obtained by PCR amplification and Sanger-mediated DNA sequencing. The positions and sequences of the sgRNA and motifs are pointed out. (C) Transcript abundance of α^D^ determined by RT-qPCR analysis in HD3 and dHD3 cells. RNA abundance for each condition (CTCF-KD or VEZF1-KD) was normalized to the scramble (SCR). (D) DNA gel electrophoresis analysis of PCR-based screening for HD3 and ΔCTCF-BS clones. (E-G) Kinetics of gene expression for the α-globin genes during erythroid maturation over a 72 h time course. Data are presented as mean ± SEM, with bars and dots representing results from at least three independent replicates. Significance was determined by an unpaired Student’s *t*-test as follows: (ns) not significant; *p < 0.05, **p < 0.01, ***p < 0.001.

We hypothesized that CTCF binds to proximal gene regions to protect regulatory elements from DNA methylation, thereby facilitating local chromatin accessibility and gene expression. To test this, we selected α^D^ as a representative gene for several reasons: α^D^ is activated in late stages of chicken embryo development, coinciding with the hemoglobin switch from fetal to adult expression [38, 39]; CTCF binds to an intergenic region between the embryonic π gene and the upstream region of α^D^ in erythrocytes from 5- and 10-day-old embryos (Figure 1A) [39, 71]; the CTCF motif is located within a DNA methylation transition zone in 10-day erythrocytes, suggesting it functions as a boundary for methylation spreading [39].

To corroborate this observation, we performed chromatin immunoprecipitation (ChIP-seq) and assay for transposase-accessible chromatin with sequencing (ATAC-seq) in immortalized chicken proerythroblasts (HD3) and their differentiated counterparts (dHD3) [72]. Unsupervised clustering of each CTCF ChIP-seq and ATAC-seq sample by principal component analysis (PCA) and correlation showed strong reproducibility across biological replicates and assays (Supplementary Data 1A-B). ATAC-seq fragment size distributions exhibited the expected nucleosomal periodicity (Supplementary Data 1C). Notably, our datasets provided superior signal-to-noise ratios, characterized by sharper ChIP-seq and ATAC-seq signals compared to existing public data for the HD3 cells (Supplementary Data 1D) [73, 74]. Differential CTCF-binding analysis revealed increased CTCF binding in dHD3 compared to HD3 cells (Supplementary Data 1E and Figure 1A). This observation supports the evidence of CTCF as a potential regulator of α^D^ gene expression in erythroid cells.

Motif analysis within the CTCF-BS region identified a CTCF motif overlapping three VEZF1 motifs (Figure 1B). VEZF1 has been previously characterized as a transcription factor (TF) that prevents DNA methylation at the 5′ end of the chicken β-globin locus [75]. Interestingly, the VEZF1 motif was also enriched within increased CTCF peaks (Figure 1B). Previous findings demonstrated that the deletion of the CTCF-BS in plasmid reporter assays resulted in a silencing effect via the spreading of DNA methylation in HD3 cells [39]. Based on these observations, we hypothesized that CTCF, VEZF1, or other regulators are required to prevent α^D^ silencing and facilitate its transcriptional activation during erythroid maturation.

**Figure 2.**
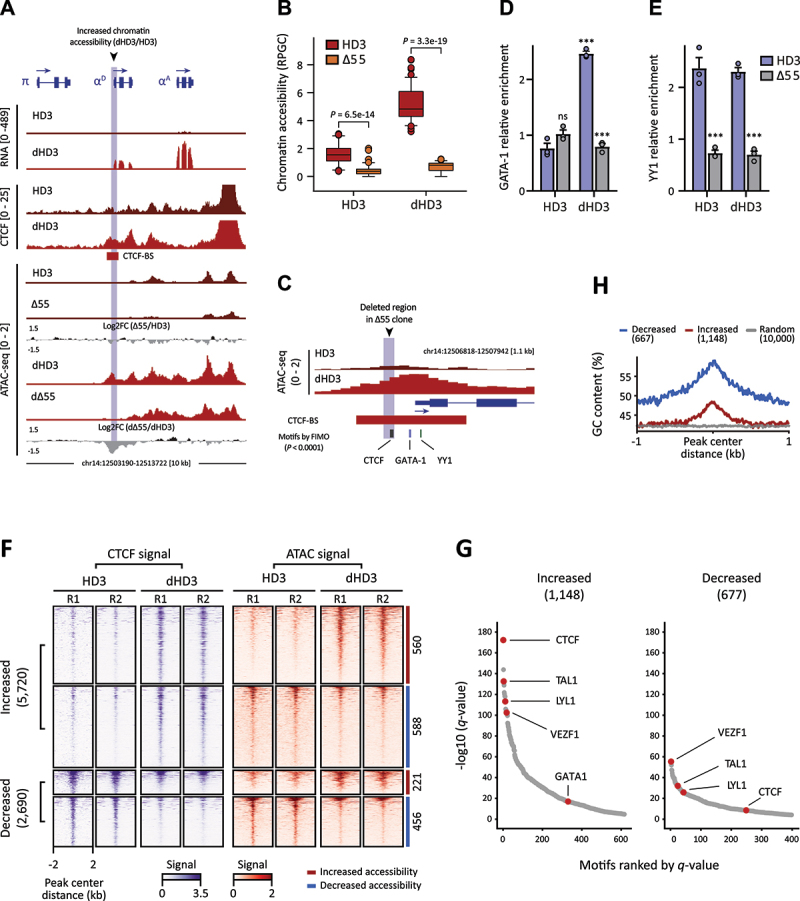
Disruption of the CTCF-BS impairs local chromatin accessibility. (A) Genomic view of the chicken α-globin locus showing signal tracks for gene expression (RNA-seq), CTCF binding (ChIP-seq), and chromatin accessibility (ATAC-seq). Differences in ATAC-seq signals are shown as log2 fold change (log2FC) of the specified comparisons. The black arrow indicates the differential accessibility site detected in HD3 relative to dHD3. (B) Box plot showing the normalized raw ATAC-seq signal counts (reads per genomic content) at the α^D^ promoter region, as highlighted in panel A. Significance (*p*-value) was determined using a two-sided Mann‑Whitney U test. (C) Binding motifs detected by FIMO are located downstream from the CTCF motif and overlapping the ATAC-seq signal around the α^D^ TSS. (D-E) YY1 and GATA-1 binding detected by ChIP-qPCR at the CTCF-BS and normalized to IgG. (F) Heatmaps of ChIP-seq for CTCF and ATAC-seq signal profiles for differentially CTCF-bound sites in undifferentiated (HD3) and differentiated (dHD3) cells. Changes in chromatin accessibility were detected by FDR < 0.05; log2FC ± 1. (G) Motif enrichment analysis of increased CTCF binding sites that exhibit differential changes in chromatin accessibility. (H) Profile plot of GC-content percentage centered on differentially CTCF-bound sites that exhibit differential changes in chromatin accessibility. Data represent the mean ± SEM of three biological replicates. Significance determined by an unpaired *t*-test as follows: (ns) not significant; *p < 0.05, **p < 0.01, and ***p < 0.001.

To test this, we performed global loss-of-function experiments by knocking down CTCF and VEZF1 in HD3 and dHD3 cells (Supplementary Data 2A and 2B). Notably, CTCF depletion (CTCF-KD), but not VEZF1 (VEZF1-KD), impaired α^D^ full activation in dHD3 (Figure 1C). To determine whether CTCF binding at this intergenic region is essential for α^D^ gene activation, we deleted its endogenous motif in HD3 cells using CRISPR-Cas9 (Figure 1B). We isolated three independent homozygous mutant clones (Δ20, Δ37, and Δ55), named according to the number of deleted base pairs (Figure 1D). Motif analysis indicated that the CTCF motif sequence was lost in the Δ37 and Δ55 clones, while all VEZF1 motifs were lost specifically in the Δ55 clone (Supplementary Data 2C). Chromatin immunoprecipitation followed by qPCR (ChIP-qPCR) revealed that CTCF binding was significantly reduced in the Δ37 and Δ55 clones but remained unchanged in the Δ20 clone relative to HD3 cells (Supplementary Data 2D). This is consistent with the specific deletions of their respective motifs. Additionally, VEZF1 binding decreased only in the Δ55 clone, corresponding to the complete loss of its binding sequences (Figure 2B).

Importantly, α^D^ gene expression was significantly reduced in the Δ37 and Δ55 clones, whereas the Δ20 clone showed no significant difference compared to HD3 (Supplementary Data 2E). Interestingly, although the Δ37 clone retained one VEZF1 binding site and maintained VEZF1 binding, this was insufficient to prevent the decrease in α^D^ expression (Supplementary Data 2E). These results confirm that the region spanning the CTCF and VEZF1 motifs is a critical regulatory element essential for α^D^ gene expression in erythroid cells.

We selected the Δ55 clone for further characterization due to its significant reduction in CTCF binding and α^D^ gene expression. The transcriptional activation of α^D^ was impaired across multiple time points of erythroid maturation induction in the Δ55 clone compared to wild-type HD3 cells, in contrast to that of the neighboring α-globin genes (π and α^D^), which remained unchanged (Figures 1E–G). Taken together, these results establish that CTCF binding at the π-α^D^ intergenic region is particularly required for α^D^ transcriptional activation during erythroid maturation. Importantly, each mutant clone exhibited a distinct pattern of lost and retained transcription factor binding motifs, suggesting that other regulators may contribute to α^D^ regulation (Supplementary Data 2F). Among the factors associated with the lost motifs, only CTCF, VEZF1, TFAP2C, HIC2, SP2, and PLAG1 are expressed in HD3 or dHD3 cells (Supplementary Data 2 G). However, an intersection analysis revealed that the CTCF motif is the only shared binding site lost in both phenotypically silenced clones (Δ37 and Δ55) (Supplementary Data 2F). This observation, combined with the fact that the transcriptional defects in the Δ55 clone mirror those of CTCF-KD cells, supports a model in which CTCF primarily mediates the regulatory activity of this intergenic region. Nevertheless, we cannot exclude the possibility of cooperative contributions from other unidentified factors.

### Perturbation of CTCF binding leads to local but not distal structural changes in the α^D^ chromatin landscape

CTCF has been shown to influence local chromatin accessibility and remodeling, thereby affecting gene expression [22, 32, 70]. To determine whether CTCF binding influences local chromatin accessibility at this locus, we performed ATAC-seq on the Δ55 clone. Following erythroid induction, we observed a significant reduction in chromatin accessibility at the CTCF-BS, extending into the α^D^ gene body, in Δ55 clone compared to dHD3 cells (Figures 2A,B). Interestingly, this decreased accessibility at the CTCF-BS was evident prior to induction (Figure 2B). These findings indicate that the CTCF-BS is essential for establishing and maintaining the accessible chromatin state necessary for the subsequent activation of the α^D^ gene during erythroid maturation.

A recent study revealed that CTCF is required for GATA-1 binding at specific sites associated with erythroid differentiation [32]. Moreover, GATA-1 and YY1 have been proposed as key transcriptional regulators of α^D^ [76, 77]. Motif analysis identified GATA-1 and YY1 downstream of the CTCF motif (Figure 2C). ChIP-qPCR assays demonstrated that GATA-1 binding increased at the CTCF-BS in dHD3 compared to HD3, whereas YY1 binding remained similar between the two conditions (Figure 2D,E). Importantly, the recruitment of both factors was significantly impaired in the Δ55 clone. These findings suggest that the CTCF-BS is required for proper recruitment of GATA-1 and YY1, which in turn facilitates chromatin accessibility and transcriptional activation of the α^D^ gene.

To explore whether the relationship between CTCF and chromatin accessibility extends to other loci, we analyzed differential chromatin accessibility at sites with increased or decreased CTCF binding in dHD3 compared to HD3 (Figure 2F). A subset of these sites displayed concordant changes in accessibility: 20.06% of sites with gained CTCF binding (1,148 of 5,720), whereas 24.79% of sites with lost binding (667 of 2,690) (Figure 2F and Supplementary Data 2G). Consistently, the CTCF motif was enriched at sites showing both increased CTCF binding and enhanced accessibility, alongside motifs for essential erythroid transcription factors such as TAL1, LYL1, and GATA-1 (Figure 2G). This observation supports our findings suggesting that the presence of lineage-specific transcription factors is crucial for stabilizing CTCF binding and vice versa, pointing toward a cooperative regulatory mechanism [78].

Consistent with previous studies, these differential CTCF binding sites were GC-rich relative to random genomic regions (Figure 2H) [69, 79]. However, it has been stated that a minor fraction of CTCF binding sites is DNA methyl-sensitive even though the presence of GCs in their motif sequences [80]. In general, CTCF binding sites represent low-methylated regions, suggesting that DNA hypomethylation may be a result of CTCF binding [7]. Altogether, these results establish a connection between CTCF and chromatin accessibility, a relationship that appears to be modulated by GC content. However, further integrative analysis using acute CTCF depletion coupled with ATAC-seq and WGBS could elucidate whether additional CTCF binding sites function as DNA methylation barriers.

**Figure 3.**
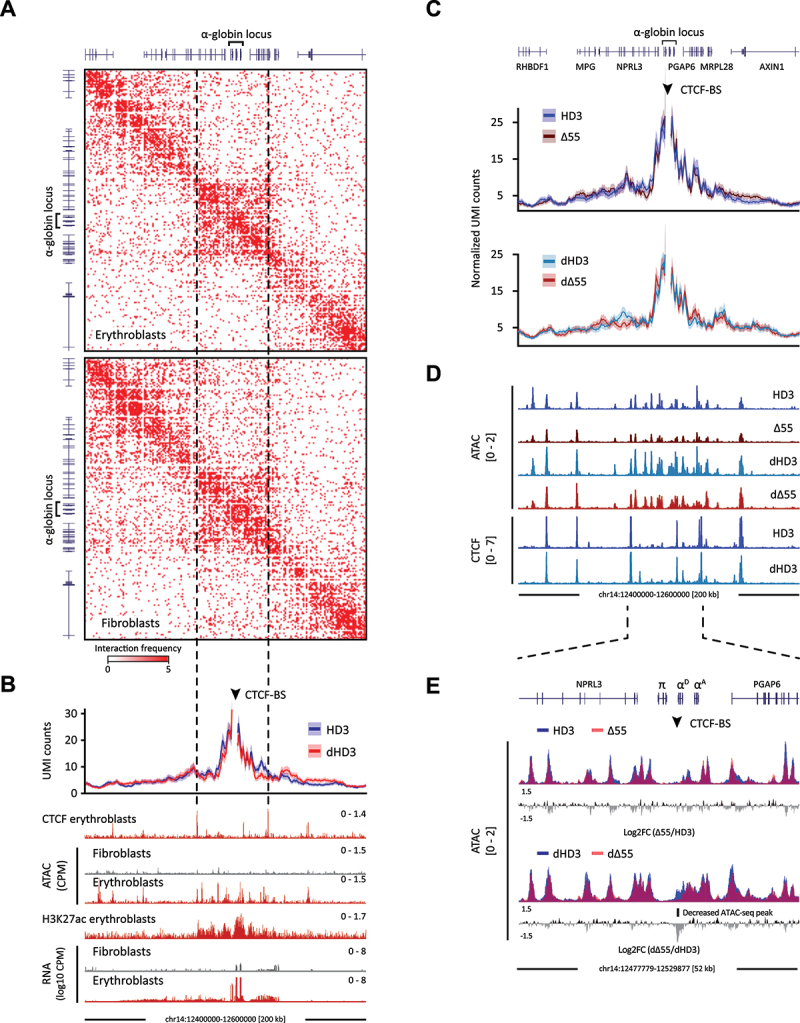
Disruption of the CTCF-BS had minimal effects on the chromatin contact profile. (A) Hi-C contact matrices showing the chicken α-globin locus in erythroblasts and fibroblasts across a 200 kb genomic window. Dotted lines demarcate the contact domain. (B) (top) CTCF-BS-centered chromatin contacts detected by UMI-4C (viewpoint). Lines show the mean of normalized UMI counts ± SD. (bottom) Genomic snapshot of CTCF, ATAC-seq, H3K27ac, and RNA-seq signals in erythroid and fibroblast. (C) UMI-4C analysis comparing α^D^ promoter interactions in HD3 and Δ55 mutants during erythroid maturation. (D) Genomic view showing the signals for ATAC-seq and CTCF binding, including the contact domain and the α-globin locus. (E) Genomic view of the contact domain showing the signals for the chromatin accessibility (ATAC-seq). Differences in ATAC-seq signals are shown as log2 fold change (log2FC) of the specified comparisons. The differential accessibility regions were computed by FDR < 0.05; |log2FC| >1.

CTCF has been extensively characterized as playing essential roles in orchestrating genome topology [20, 81, 82]. By analyzing chromatin contacts derived from public Hi-C, we observed that the α^D^ gene is structured into a contact domain spanning approximately 52 kb, including the neighboring genes *NPRL3*, π (*HBZ*), α^A^ (*HBA1*), and *PGAP6* (Figure 3(A)) [74]. Intriguingly, this contact domain was detected in both chicken erythroblasts and fibroblasts, suggesting that the organization of this locus is not restricted to the erythroid cell lineage. Thus, this structural organization may facilitate the communication between regulatory elements and their target genes [83].

To explore the mechanism of CTCF in controlling α^D^ gene transcription via its structural functions, we profiled CTCF-BS-centered contacts by UMI-4C (Figure 3B and Supplementary Data 4A). We did not detect significant changes in chromatin contacts when comparing HD3 and dHD3 cells. It is worth noting that, most interactions were restricted within the contact domain demarcated by CTCF-binding sites in chicken erythroblasts. Additionally, analysis of public data for ATAC-seq and histone H3K27ac revealed a high chromatin accessibility signal coinciding with histone H3K27ac deposition within this contact domain in erythroblasts [74, 84]. Similarly, genes within this contact domain exhibit a higher RNA signal than those outside. In contrast, chromatin accessibility and α-globin expression were reduced in fibroblasts (Figure 3B). Altogether, these data suggest that the active CREs of the α-globin locus exploit three-dimensional proximity, which may facilitate the transcriptional activation of the α-globin locus during erythropoiesis.

Remarkably, UMI-4C analysis revealed no significant differences in the chromatin contact profiles centered at the CTCF-BS between the Δ55 clone and HD3 during erythroid maturation induction (Figure 3C). While UMI-4C provides quantitative, high-resolution profiling of chromatin contacts from a specific viewpoint, we acknowledge that single-viewpoint approaches may miss subtle structural perturbations involving other regulatory elements. Nevertheless, the lack of detectable changes in contacts centered at the CTCF-BS implies that the CTCF-BS functions as a local CRE for the α^D^ gene rather than as a structural anchor or architectural element mediating long-range interactions to the α^D^ promoter. To further support this hypothesis, we integrated ATAC-seq data focused on the contact domain region (Figure 3D and Supplementary Data 4B). By computing differences in ATAC-seq signals, we do not detect significant differences in chromatin accessibility at distal regions from the CTCF-BS in the Δ55 clone and HD3, both in the undifferentiated and differentiated conditions (Figure 3E). This suggests that CTCF-BS is primarily required to modulate local chromatin accessibility of the α^D^ promoter, rather than functioning as a distal regulatory element or as a facilitator of distal interactions.

### CTCF prevents the spreading of DNA methylation towards the α^D^ gene

During erythroid maturation, DNA displayed global hypomethylation at regions with increased chromatin accessibility [27, 85]. This DNA hypomethylation is directly linked to the DNA-binding activities of regulatory proteins, including CTCF [7, 86]. We hypothesized that the CTCF binding sites coordinate local DNA methylation and chromatin accessibility to guarantee α^D^ gene transcriptional activation.

**Figure 4.**
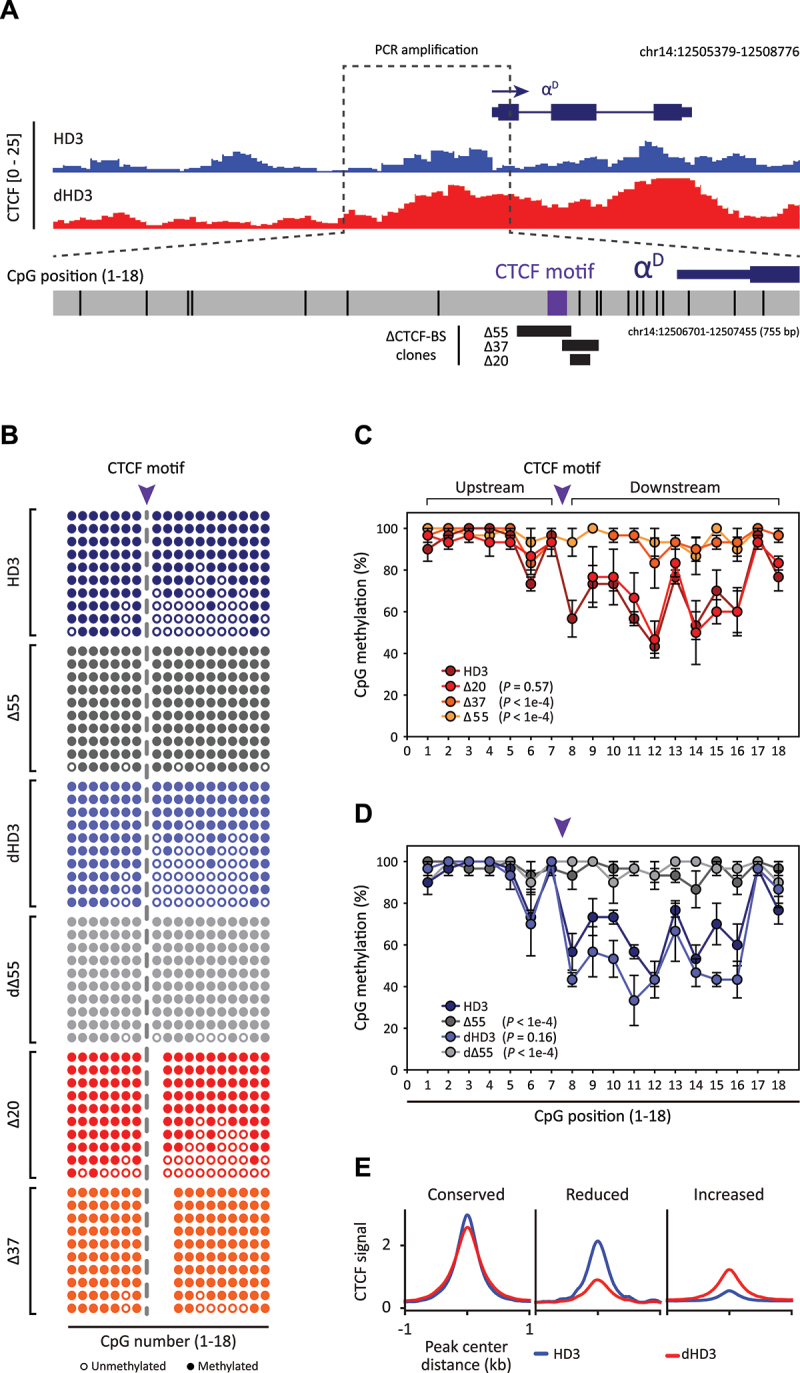
The CTCF-BS prevents DNA methylation from spreading towards the α^D^ gene in erythroid cells. (A) Genomic view of the PCR-amplified region used for bisulfite conversion analysis at the CTCF-BS region. CpG positions (1–18) relative to the CTCF motif are shown as vertical lines. (B) The methylation status of individual CpG sites is displayed as filled circles (methylated) and open circles (unmethylated) in each condition. Missing circles represent deleted CpGs in the mutant clones. (C and D) Mean methylation percentages for each CpG in mutant and HD3 cells. Data represent the mean ± SEM from three independent bisulfite conversion experiments (8–10 sequenced clones per experiment). Significance was determined by a two-tailed unpaired Student *t*-test. (E) Profile plots showing the CTCF-binding signals at differentially CTCF-bound sites in HD3 and dHD3 conditions.

A previous study revealed that the CTCF motif within the CTCF-BS is located in a DNA methylation transition zone in the intergenic region between π and α^D^ genes. Furthermore, deletion of the CTCF motif was shown to increase DNA methylation toward the α^D^ gene *in vitro* [39]. Based on this observation, we analyzed the methylation status of 18 CpGs spanning the CTCF motif at this intergenic region using bisulfite conversion followed by PCR amplification and sequencing. Notably, the CTCF motif is positioned between CpG 7 and 8 (Figure 4A).

In HD3 cells, ~67% of downstream CpGs (8–18) were methylated, whereas the Δ37 and Δ55 clones exhibited significantly higher methylation levels (~95%) in undifferentiated cells (Figure 4B,C). In contrast, the Δ20 clone showed an intermediate methylation level (~78%), more closely resembling the HD3 profile. These results suggest that the CTCF-BS is essential to restrict the spread of DNA methylation toward the α^D^ gene.

To further validate this mechanism, we assessed DNA methylation during erythroid maturation. While the HD3 cells underwent significant demethylation during this process, the Δ55 clone maintained high methylation levels (~95%) downstream of the CTCF-BS (Figure 4D). The findings indicate that the CTCF-BS serves as a DNA methylation barrier at this intergenic region, protecting the promoter-proximal region from upstream silencing marks to ensure α^D^ gene activation.

Acute depletion of CTCF has shed light on the multiple functions of CTCF, revealing two classes of CTCF-binding sites: high-affinity sites function as structural regulators of chromatin topology, whereas low-affinity sites are involved in transcriptional regulation [20, 81, 87, 88]. Notably, conserved CTCF-binding sites exhibit a stronger enrichment signal than increased sites during erythroid maturation (Figure 4E). This suggests that the increased sites may act as transcriptional regulators, similar to the CTCF-binding site in the π-α^D^ intergenic region. Our findings indicate that CTCF directly regulates α^D^ transcription by modulating the local chromatin state. This is achieved by facilitating the binding of transcriptional regulators such as GATA-1 and YY1, and by preventing the propagation of DNA methylation from the intergenic region.

### A CTCF-BS protects the HBA2 gene from DNA methylation in human erythroid cells

**Figure 5.**
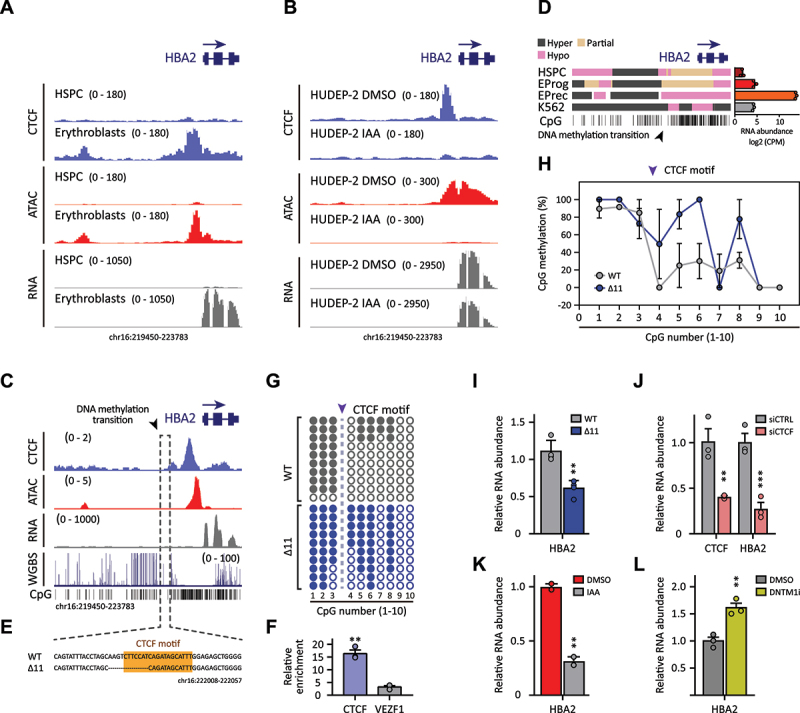
A CTCF-BS prevents DNA methylation from spreading towards the human *HBA2* gene. (A) Genomic view of the human *HBA2* locus showing the signals for CTCF, ATAC-seq, and RNA-seq in: hematopoietic progenitor and stem cells (HPSC) and erythroblasts; HUDEP-2 cells control (DMSO) and treated with auxin (IAA) to acute CTCF depletion (B). (C) Genomic snapshot showing the signals for CTCF, ATAC-seq, RNA-seq, and WGBS. The DNA methylation transition zone is highlighted in dotted lines. (D) DNA methylation segments identified by WGBS and visualized from the ChIP-Atlas [92]. (E) Pairwise DNA alignment showing the sequences of the WT and Δ11 clone. Sequences were obtained by PCR amplification and Sanger-mediated DNA sequencing. (F) ChIP-qPCR analysis of CTCF and VEZF1 enrichment at the DNA methylation transition zone in K562 cells. (G) Methylation status of individual CpGs represented as follows: filled circles (methylated) and open circles (unmethylated). The dashed line indicates the position of the mutated CTCF motif. (H) DNA methylation percentages for each CpG in wild-type and ΔCTCF-BS in K562 cells. Data represent the mean ± SEM from two independent bisulfite conversion experiments (WT: *n* = 24 clones total, 10 and 14 clones per replicate; Δ11: *n* = 19 clones total, 9 and 10 clones per replicate). (I) *HBA2* gene abundance determined by RT-qPCR analysis in the Δ11 compared with the WT. (J) HBA2 gene expression measured by RT-qPCR in control (siCTRL) and CTCF knockdown (siCTCF) K562 cells. Data were normalized to the WT or siCTRL condition. (K-L) Relative RNA abundance of *HBA2* detected by RNA-seq analysis in K562 cells under control (DMSO), acute CTCF depletion (IAA), and DNMT1 methyltransferase inhibitor (DNMT1i). Data represent the mean ± SEM of at least three experimental replicates. Significance was determined by a two-tailed unpaired Student *t*-test as follows: *p < 0.05, **p < 0.01, and ***p < 0.001.

The structural role of CTCF in shaping chromatin architecture is evolutionarily conserved across vertebrates [71, 89–91]. We hypothesized that the barrier function of the CTCF-binding site (CTCF-BS) against DNA methylation might also be conserved. To test this, we examined the human ortholog HBA2. Analysis of public ChIP-seq, ATAC-seq, and RNA-seq data across human erythropoiesis revealed that CTCF binds the HBA2 promoter in erythroblasts, but not in hematopoietic stem and progenitor cells (HSPCs) (Figure 5A) [30]. This increament in CTCF binding correlated with enhanced chromatin accessibility and the upregulation of HBA2 transcription. Furthermore, acute depletion of CTCF in the erythroid progenitor cell line HUDEP-2 resulted in decreased HBA2 expression (Figure 5B) [32]. These data indicate that, similar to the α^D^ locus, CTCF is essential for maintaining chromatin accessibility and ensuring HBA2 transcriptional activation in human erythroid cells, suggesting a conserved regulatory mechanism in vertebrates.

To further investigate this mechanism, we analyzed public CTCF ChIP-seq and whole-genome bisulfite sequencing (WGBS) data from human K562 erythroleukemic cells [93, 94]. We observed a DNA methylation transition zone upstream of the CTCF-BS, characterized by hypermethylated CpGs upstream and hypomethylated CpGs downstream (Figure 5C). Visualization of the methylation status revealed that this transition zone is partially methylated towards the *HBA2* gene in HSPCs and erythroid progenitors (EProg), whereas it is hypomethylated in erythroblasts (EPrec) and K562 cells (Figure 5D). Notably, this transition occurs within a CpG-rich region, suggesting that DNA methylation levels are dynamically modulated during erythropoiesis [95]. Interestingly, this DNA methylation pattern coincides with *HBA2* RNA abundance.

Motif analysis identified a CTCF consensus sequence within this methylation transition zone. ChIP-qPCR confirmed the enrichment of CTCF, but not VEZF1, at this site (Figure 5F). Using CRISPR-Cas9, we generated an 11-bp deletion (Δ11) at the CTCF motif and isolated a representative cell clone (Figure 5E). We then assessed the impact of this mutation on the DNA methylation profile of the HBA2 gene using bisulfite sequencing. Similar to our observations in HD3 cells, the Δ11 clone exhibited an approximately 44% increase in CpG methylation downstream of the CTCF motif compared to wild-type (WT) cells (Figures 5G,H). This gain in DNA methylation was associated with a significant reduction in HBA2 transcription (Figure 5I). Additionally, CTCF knockdown in K562 cells led to reduced HBA2 transcript levels (Figure 5J). Analysis of public RNA-seq data confirmed that acute CTCF depletion results in HBA2 downregulation [96], while inhibition of DNA methyltransferase 1 (DNMT1) activity enhanced *HBA2* expression (Figure 5K,L) [97]. Therefore, the connection between DNA methylation and CTCF binding seems to be regulated through the CTCF-BS sequence positioned upstream from the *HBA2* gene.

**Figure 6.**
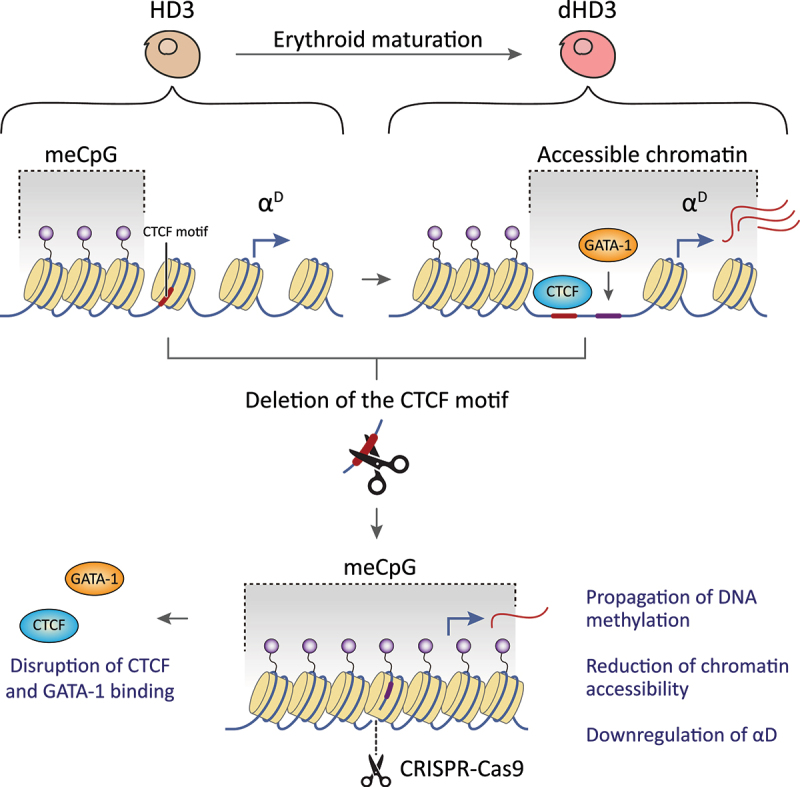
The CTCF-BS acts as a functional barrier against the spreading of DNA methylation in erythroid cells. Graphical abstract illustrating the proposed model in which the CTCF binding site (CTCF-BS) serves as a boundary element, preventing the propagation of DNA methylation toward the α^D^ gene. *In vivo* perturbation of the CTCF-BS via CRISPR-Cas9-mediated deletion in chicken erythroid cells resulted in reduced chromatin accessibility, the loss of CTCF and GATA-1 binding, spreading of DNA methylation, and downregulation of α^D^ expression.

Altogether, these results suggest that the CTCF-BS regulates *HBA2* transcription by modulating DNA methylation deposition. This may represent a conserved mechanism across vertebrates, including chicken and human erythroid cells (Figure 6). We proposed that a group of increased CTCF binding sites during cell differentiation modulates chromatin accessibility and prevents DNA methylation spreading at *cis* regulatory elements to ensure gene transcriptional activation.

## Discussion

Fine-tuning gene expression during cell differentiation is mediated by transcriptional regulators acting on their target sequences within *cis*-regulatory elements (CREs), such as promoters, enhancers, and insulators [98]. Nevertheless, CTCF has been linked to modulating local DNA methylation deposition at CREs [7, 86]. In this study, we profiled the CTCF binding landscape across chicken erythroid maturation using immortalized proerythroblasts (HD3), analyzing its dynamics and role in gene regulation during cell differentiation.

The regulatory functions of CTCF are conserved across vertebrates [20, 40, 70, 81]. Given this conservation, it is likely that shared mechanisms of transcriptional activation or repression operate across species. For instance, the insulator function of CTCF has been documented in several organisms, including human, mouse, and chicken [13, 20, 24, 87, 99, 100]. In addition to the insulator function in restricting enhancer-promoter communications, the role of CTCF in shaping local DNA methylation deposition has been explored in vertebrates [7, 101–103]. For instance, we previously demonstrated that the CTCF-BS delimits a DNA hypermethylated region from an unmethylated transition region in erythrocytes from 10-day-old embryos coinciding with the hemoglobin switching, where the embryonic π gene is silenced and the adult α^D^ gene is activated. Moreover, the sequence of the CTCF binding motif in this region is essential to maintain an unmethylated state toward the α^D^ gene *in vitro*, indicating that the CTCF-BS represents a DNA sequence functioning as a barrier to prevent the spreading of DNA methylation [39].

The α-globin orthologs α^D^ (*HBAD*) in chicken and *HBA2* in human represent a hallmark of erythroid-specific abundant genes associated with erythroid maturation. Perturbation of CTCF binding at these gene promoters resulted in reduced chromatin accessibility, DNA methylation spreading, and reduced gene expression, suggesting a CTCF-dependent conserved regulatory mechanism. Further analysis revealed that this effect is independent of the architectural function of CTCF, as no significant alterations in chromatin structure were observed. Additional regulatory roles of CTCF beyond its structural function have been documented [104,105]. For instance, CTCF can modulate local DNA methylation through two distinct mechanisms: either by its poly(ADP-ribosyl)ation, which inhibits the deposition of DNA methyltransferases, or by directly recruiting TET methylcytosine dioxygenase (TET) enzymes to promote active demethylation [106–108]. Consistent with these functions, we determined that CTCF binding at the π-α^D^ intergenic region is required to prevent DNA methylation spreading towards the α^D^ gene promoter. The mechanism underlying this protective role requires further experimental investigation.

Our findings are particularly relevant to the globin switch that occurs during chicken embryo development. DNA methylation-mediated silencing of the embryonic π gene extends precisely to the CTCF-bound regulatory element characterized in this study, where CTCF is constitutively bound in both 5-day and 10-day red blood cells (5dRBCs and 10dRBCs). This observation supports a model in which this regulatory element functions as a barrier that protects the adult α^D^ and α^A^ genes from the spread of DNA methylation-mediated silencing during erythroid maturation, ensuring proper temporal regulation of gene expression during development.

We observed a strong correlation between CTCF-BS deletion, increased DNA methylation, and reduced α^D^ expression, though formal causality remains to be established. Acute depletion of CTCF (e.g., using mAID or dTAG systems) followed by analysis of DNA methylation and chromatin accessibility at this locus would provide valuable evidence to further support the role of CTCF in preventing silencing. The DNMT1 inhibitor data in K562 cells (Figure 5L) partially supports a causal role for DNA methylation in gene repression. Future experiments using DNMT inhibitors (e.g., 5-azacytidine) or dCas9-TET1-mediated locus-specific demethylation could test whether DNA methylation directly represses α^D^ transcription. Furthermore, both reduced chromatin accessibility (Figure 2B) and DNA methylation spreading (Figure 4B) are present in the undifferentiated Δ55 clone, raising questions about their temporal hierarchy. While we hypothesize that DNA hypermethylation at the downstream CpGs precedes chromatin compaction, recent studies indicate that chromatin accessibility and DNA methylation can exhibit discordant temporal dynamics during differentiation [109, 110]. Time-resolved analysis will be necessary to resolve this order.

Genome-wide profiling of CTCF occupancy during erythroid maturation revealed a subset of binding sites that exhibited low enrichment but showed increased chromatin accessibility. Also, these sites were enriched in CTCF and GATA-1 motifs, indicating a possible cooperativity between CTCF and erythroid regulators in modulating the chromatin landscape. This observation is supported by our data and recent findings, in which CTCF binding facilitates the recruitment of key cell-type-specific TFs, including the master erythroid regulator GATA-1 [32]. While HD3 cells represent a well-characterized model for studying erythroid differentiation, we acknowledge that their transformed nature may not fully recapitulate the epigenomic landscape of primary erythroid cells, though future studies in primary cells would further strengthen our conclusions.

Our study provides additional insights into the regulatory function of CTCF beyond its architectural function. Taking into account our results for the α^D^ and HBA2 loci, we propose that dynamic CTCF-binding sites, characterized by increased chromatin accessibility and high GC-content, may represent DNA sequences functioning as barriers to prevent the deposition and spreading of DNA methylation towards lineage-specific genes during development and cell differentiation. This model is supported by observations in other systems. CTCF has been shown to function as a barrier insulator between the tandem genes BLU and RASSF1A, establishing distinct epigenetic domains and preventing the spread of heterochromatin-associated methylation [111]. Furthermore, CTCF loss in cancer cells leads to spatially distinct DNA hypermethylation events at CTCF binding sites, and forced downregulation of CTCF results in gene silencing that can be reversed by DNA methylation inhibition [12]. These observations support the broader applicability of CTCF-mediated methylation barriers as a conserved mechanism for protecting tissue-specific genes from silencing during development and in disease contexts.

## Supplementary Material

Supplemental Material

## Data Availability

The raw and processed data supporting the findings of this study are openly available in Gene Expression Omnibus at https://www.ncbi.nlm.nih.gov/geo/, reference numbers (GSE296417, GSE296376, and GSE296416), and Zenodo at https://doi.org/10.5281/zenodo.21145702.
