# Supplementary material for: CTCF delimits a DNA methylation transition at the intergenic region between the embryonic and adult α-globin genes
Source: Epigenetics. 2026 Aug 3;21(1):2711190. doi: 10.1080/15592294.2026.2711190 (PMC13436835; doi:10.1080/15592294.2026.2711190)
Supplement: Supplementary file 1 [file KEPI_A_2711190_SM0439.docx]

**Appendix**

Table 1

*Means, standard deviations, and sample sizes for overall T-SEL and respective sub-area.*

| **Constructs** | ***N*** | **Min** | **Max** | **Std Dev** | **Var** | **Mean** |
| --- | --- | --- | --- | --- | --- | --- |
| Self-Awareness | **5** | **2** | **4** | **0.71** | **0.50** | **3.40** |
| Relationship Skills | **5** | **2** | **4** | **0.79** | **0.62** | **3.12** |
| Cultural Awareness | **5** | **1** | **4** | **0.81** | **0.66** | **3.08** |
| Social Awareness | **5** | **2** | **4** | **0.79** | **0.62** | **3.04** |
| Self-Management | **5** | **2** | **4** | **0.82** | **0.67** | **3.00** |
| Responsible Decision-Making | **5** | **2** | **4** | **0.73** | **0.53** | **2.88** |
| ***Overall T-SEL*** | **5** | **1** | **4** | **0.82** | **0.68** | **3.07** |

Table 2

*T-SEL Sub-Area Response Summary*

| **T-SEL Sub Areas** | **Min** | **Max** | **Mean** | **Std Dev** | **Var** | ***N*** |
| --- | --- | --- | --- | --- | --- | --- |
| **Self-Awareness** | | | | | | |
| Use body language and non-verbal cues effectively to respond to others. | 2 | 4 | 3.20 | 0.84 | 0.70 | 5 |
| Able to use tone of voice to convey emotions and meaning effectively. | 2 | 4 | 3.20 | 0.84 | 0.70 | 5 |
| Use of listening skills when communicating with others. | 3 | 4 | 3.60 | 0.55 | 0.30 | 5 |
| Communicate needs in constructive ways. | 3 | 4 | 3.40 | 0.55 | 0.30 | 5 |
| Demonstration of a sense of personal purpose, knowing life is important and meaningful. | 2 | 4 | 3.60 | 0.89 | 0.80 | 5 |
| **Overall Self-Awareness** | **2** | **4** | **3.40** | **0.71** | **0.50** | **25** |
| **Relationship Skills** | | | | | | |
| Able to easily make new friends with peers. | 2 | 4 | 3.20 | 0.84 | 0.70 | 5 |
| Able to maintain long-term friendships with peers. | 2 | 4 | 3.00 | 0.71 | 0.50 | 5 |
| Choose positive interactions when engaging with friends inside the class. | 2 | 4 | 3.20 | 0.84 | 0.70 | 5 |
| Choose positive interactions when engaging with friends during free-time. | 2 | 4 | 3.20 | 0.84 | 0.70 | 5 |
| Demonstrate honesty and integrity when interacting with peers, insisting on fairness in constructive ways. | 2 | 4 | 3.00 | 0.71 | 0.50 | 5 |
| **Overall Relationship Skills** | **2** | **4** | **3.12** | **0.79** | **0.62** | **25** |
| **Cultural Awareness** | | | | | | |
| Work cooperatively with a diverse group of peers. | 3 | 4 | 3.40 | 0.55 | 0.30 | 5 |
| Respect and celebrate peer differences. | 3 | 4 | 3.40 | 0.55 | 0.30 | 5 |
| Able to use strategies to express themselves effectively in different cultural contexts. | 2 | 4 | 3.20 | 0.84 | 0.70 | 5 |
| Identify personal characteristics, habits, or biases and show an understanding of how those may affect others across cultures. | 1 | 4 | 2.60 | 1.14 | 1.30 | 5 |
| Advocate productively for themselves or others when situations are not equitable. | 2 | 4 | 2.80 | 0.84 | 0.70 | 5 |
| **Overall Cultural Awareness** | **1** | **4** | **3.08** | **0.81** | **0.66** | **25** |
| **Social Awareness** | | | | | | |
| Exhibit patience and understanding when communicating with others. | 2 | 4 | 3.20 | 0.84 | 0.70 | 5 |
| Able to navigate and solve problems caused by differences in opinions. | 2 | 4 | 2.80 | 0.84 | 0.70 | 5 |
| Display the ability to understand the perspectives of people in social situations. | 2 | 4 | 3.20 | 0.84 | 0.70 | 5 |
| Capable of effectively regulating emotions to suit the demands of a given social situation. | 2 | 3 | 2.60 | 0.55 | 0.30 | 5 |
| Present a positive attitude towards their peers when working through a difficult task. | 3 | 4 | 3.40 | 0.55 | 0.30 | 5 |
| **Overall Social Awareness** | **2** | **4** | **3.04** | **0.79** | **0.62** | **25** |
| **Self-Management** | | | | | | |
| Able to limit outbursts when engaged in activities. | 3 | 4 | 3.40 | 0.55 | 0.30 | 5 |
| Able to confidently engage in group activities. | 3 | 4 | 3.60 | 0.55 | 0.30 | 5 |
| Able to perform well on academic tasks. | 2 | 3 | 2.20 | 0.45 | 0.20 | 5 |
| Able to confront a problem when assigned a difficult task. | 2 | 4 | 2.80 | 0.84 | 0.70 | 5 |
| Able to set and accomplish personal goals. | 2 | 4 | 3.00 | 0.71 | 0.50 | 5 |
| **Overall Self-Management** | **2** | **4** | **3.00** | **0.82** | **0.67** | **25** |
| **Responsible Decision-Making** | | | | | | |
| Identify choices through critical thinking that result in the best outcomes for all involved. | 2 | 4 | 2.80 | 0.84 | 0.70 | 5 |
| Demonstrate understanding that leadership skills can be learned and explored in ways that are important to them. | 2 | 4 | 3.20 | 0.84 | 0.70 | 5 |
| Identify distractions and apply simple solutions to overcome them. | 2 | 3 | 2.60 | 0.55 | 0.30 | 5 |
| Resist impulses to maintain appropriate personal **physical** boundaries with others. | 2 | 4 | 2.80 | 0.84 | 0.70 | 5 |
| Resist impulses to maintain appropriate personal **emotional** boundaries with others. | 2 | 4 | 3.00 | 0.71 | 0.50 | 5 |
| **Overall Responsible Decision Making** | **2** | **4** | **2.88** | **0.73** | **0.53** | **5** |

*Figure 1.* Standard SWOT Analysis Table *[Adapted from Leigh (2010, p. 116)]*

Qualtrics Survey
